# Next-generation dental implant surface modifications for osseointegration: From nanoengineering to clinical translation

**DOI:** 10.1177/03000605261489355

**Published:** 2026-09-20

**Authors:** Sukumaran Anil, Vishnupriya K Sweety, Anjali Mol, Aman Singh, Namrata Suresh, Nandita Suresh

**Affiliations:** 1 Center of Excellence in Precision Medicine and Digital Health, Department of Physiology, Faculty of Dentistry, 26683 Chulalongkorn University, Thailand; 2 Dental Research Cell, Dr. D. Y. Patil Dental College and Hospital, Dr. D. Y. Patil Vidyapeeth, India; 3 29352 Government Medical College, Pathanamthitta, India; 4 Department of Oral Medicine and Radiology, Manipal College of Dental Sciences, 29228 Manipal Academy of Higher Education, India; 5 Department of Oral and Maxillofacial Diseases, Helsinki University and University Hospital, Finland

**Keywords:** Dental implant surface modification, osseointegration, titanium dioxide nanotube arrays, bioactive coatings, osteoimmunomodulation, peri-implantitis, antimicrobial surfaces, additive manufacturing, narrative review

## Abstract

This narrative review, prepared in accordance with the Scale for the Assessment of Narrative Review Articles, surveys next-generation dental implant surface modifications for osseointegration, from nanoengineering to clinical translation. Peri-implantitis affects approximately 19.53% of patients and 12.53% of implants. Because peri-implant tissue destruction reflects a dysregulated host inflammatory response to an unavoidable, lifelong microbial challenge rather than bacterial colonization alone, the design target is shifting from surfaces that are simply bactericidal to surfaces that regulate inflammation while supporting bone integration. Surface design has evolved from machined titanium through sandblasted and acid-etched (SLA) platforms and the superhydrophilic SLActive surface to nanoengineered, bioactive, and stimuli-responsive architectures. SLActive surfaces prevent hydrocarbon adsorption so that near-zero water contact angles are retained, whereas unprotected SLA surfaces progressively lose hydrophilicity with storage time. Electrochemically anodized titanium dioxide nanotube arrays elicit diameter-dependent cellular responses. Tubes with diameters of 70 to 100 nm drive approximately 10-fold elongation of mesenchymal stem cells and selective osteogenic differentiation and enhance bone bonding 9-fold in vivo. Bioactive coatings incorporating nanostructured hydroxyapatite, therapeutic ion-doped bioactive glasses (niobium, cerium, and zinc), and immobilized growth factors such as bone morphogenetic protein 2 and vascular endothelial growth factor actively direct cellular behavior. Antimicrobial strategies, including silver nanoparticles, antimicrobial peptides (GL13K and HHC-36), and photodynamic or photothermal therapies, address microbial colonization while preserving osteogenic potential. However, ensuring lifelong efficacy remains the key challenge for any release-based approach. Stimuli-responsive systems employ pH-responsive gating, zeolitic imidazolate framework-8 metal–organic framework carriers, and enzyme-cleavable linkers to enable microenvironment-triggered release. Additive manufacturing advances, including triply periodic minimal surface lattices and four-dimensional (4D)-printed shape-memory nickel–titanium constructs, enable patient-specific geometries with tailored porosity gradients. In contrast, femtosecond laser texturing, plasma treatments, and atomic layer deposition provide atomic- to micro-scale control. Emerging graphene oxide derivatives, modified polyetheretherketone, and zirconia-based ceramics further broaden the design landscape. Despite robust preclinical evidence, translational progress remains constrained by regulatory complexity, manufacturing scalability, and limited long-term clinical data. Herein, we synthesize preclinical and clinical evidence, identify research gaps, propose artificial intelligence-guided design directions, and outline pathways for translating next-generation surfaces into routine practice. We explicitly outline the limitations inherent to a narrative synthesis.

## Graphical abstract

**Graphical abstract:**
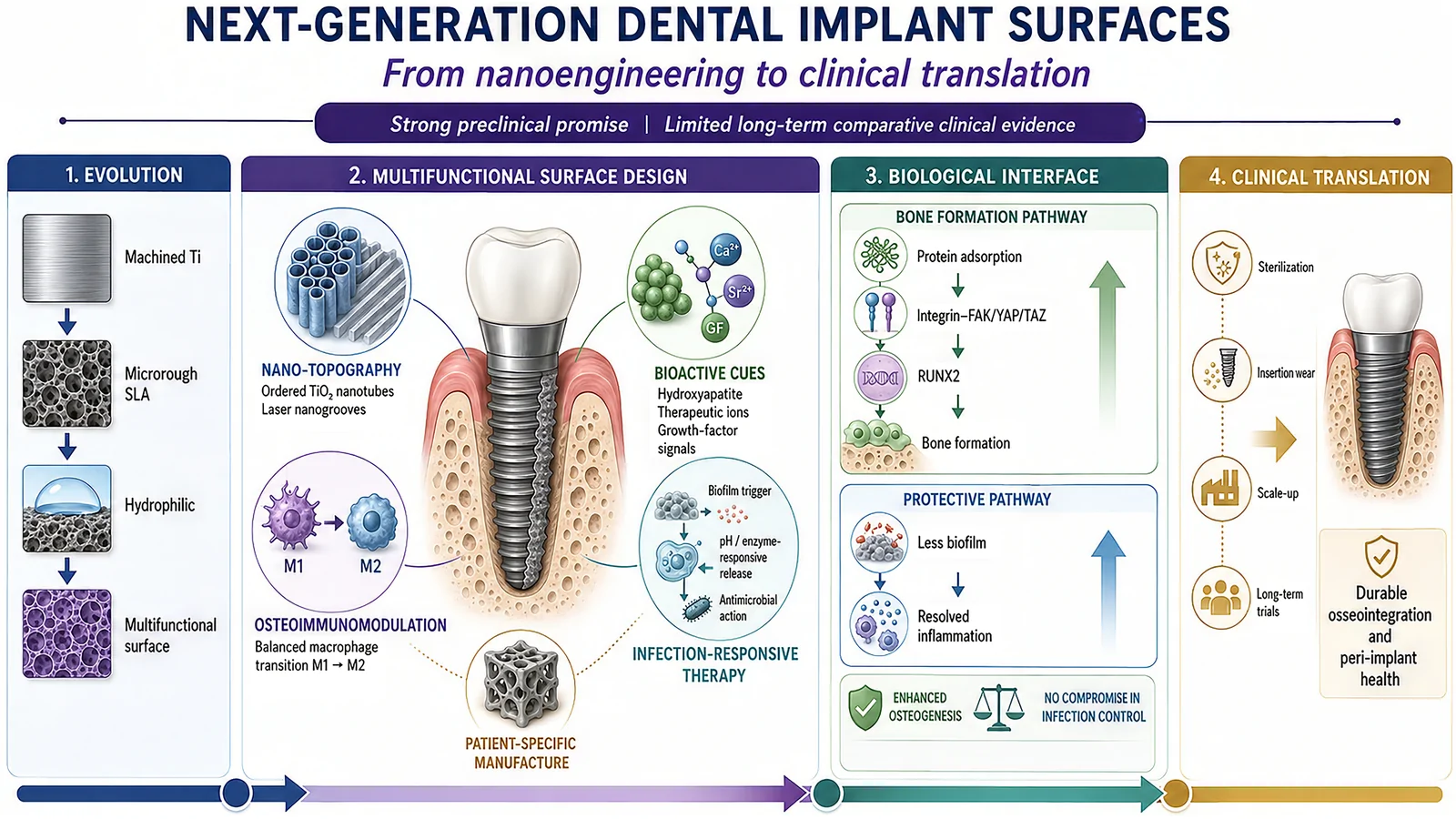
This is a visual representation of the abstract.

## Introduction

The field of dental implantology has transformed remarkably since Per-Ingvar Brånemark’s accidental discovery of osseointegration in 1952. While conducting experiments on bone healing using titanium optical chambers implanted in rabbit tibiae, Brånemark observed that titanium bonded inseparably to the surrounding bone tissue. He termed this phenomenon as “osseointegration,” defining it as a direct structural and functional connection between living, ordered bone and the surface of a load-bearing implant.^1^ This unexpected finding laid the groundwork for modern implant dentistry, leading to the first successful human dental implant placement in 1965 and the influential 1982 Toronto Osseointegration Conference, which established implantology as a cornerstone of restorative dentistry.^2,3^ Osseointegration forms the biological basis for implant success. It involves a complex series of cellular and molecular events that mirror natural bone fracture healing, beginning with protein adsorption onto the implant surface, followed by the recruitment, proliferation, and differentiation of osteoprogenitor cells guided by specific signaling pathways.^4^ Successful osseointegration achieves implant survival rates exceeding 95% at 5 years, as supported by established surface-modification technologies.^5^

Despite these successes, significant clinical challenges remain. Peri-implantitis, the inflammatory destruction of peri-implant tissues with progressive bone loss, affects approximately 19.53% of patients (95% confidence interval (CI): 12.87%–26.19%) and 12.53% of implants according to a systematic review and meta-analysis of 57 studies.^6^ More recent analyses applying the 2017 World Workshop classification criteria report even higher prevalence, with peri-implant mucositis affecting 63.0% and peri-implantitis affecting 25.0% of patients.^7^ Risk factors, including a history of periodontitis (odds ratio (OR) = 3.84), cigarette smoking (relative risk (RR) = 2.07), diabetes mellitus, and poor oral hygiene, significantly increase susceptibility to these complications.^8^ These figures are conventionally read as a mandate for antimicrobial surfaces, but that reading deserves qualification. Every surface exposed to the oral environment, including the abutment and the transmucosal implant neck, is colonized by bacteria within hours of exposure, and this colonization is unavoidable and lifelong. The permanent host–microbe confrontation at the transmucosal interface produces a continuous inflammatory environment. Disease supervenes when pro-inflammatory mechanisms outweigh those that resolve inflammation, for reasons that include host genetic makeup, smoking, glycemic control, and plaque control.^9^ On this reading, the destruction that defines peri-implantitis is driven by an uncontrolled host response rather than by bacterial presence as such.^10^ Two consequences follow for surface design. First, the therapeutic target should include surfaces that moderate or resolve inflammation, not only surfaces that kill bacteria. Second, any antimicrobial strategy based on elution of drugs or ions must be judged against a service life measured in decades, which no finite drug or ion reservoir can meet. Contact-killing and nonfouling chemistries are more plausible in principle, but they too must demonstrate lifelong efficacy in the mouth. We therefore use the term antimicrobial throughout this review in the restricted sense of activity demonstrated over a defined observation period, and we distinguish it explicitly from a claim of durable, lifelong sterility of the implant surface.

Implant surfaces have evolved through distinct generations, each reflecting advances in understanding of the bone–implant interface. First-generation machined surfaces, characterized by relatively smooth, turned titanium, provided the foundation established by Brånemark's original work but required extended healing periods of 3–6 months before loading. The 1980s introduced rougher, microporous surfaces produced by plasma-spraying techniques, which accelerated bone ingrowth but posed challenges for coating adhesion and long-term stability.^11^ The 1990s brought moderately rough surfaces. The decisive evolutionary step was not sandblasting but the microrough topography generated by acid attack, whose characteristic peak-to-peak distance is well below cell dimensions and drives mesenchymal cells toward osteogenic differentiation rather than proliferation and a fibroblast-like phenotype, while promoting platelet activation and contact osteogenesis.^12^ Sandblasting with large grit followed by acid etching (SLA) is the most successful expression of this approach and remains a benchmark, demonstrating 10-year survival rates of 98.8%–99.7%.^13,14^ The dual acid-etched (DAE) osseotite surface, introduced by Implant Innovations Inc. without a sandblasting step, was an equally prominent expression of the same principle and provided much of the histological evidence base for contact osteogenesis on microrough titanium.^15,16^ SLActive surfaces, introduced by Straumann in 2005, further enhanced outcomes by preserving a chemically active, hydrocarbon-free titanium surface in protective storage, maintaining near-zero water contact angles until placement. The water contact angle of any titanium surface is not a fixed material attribute. It is determined jointly by topography, which is fixed, and by surface chemistry, which is not, because high-energy titanium progressively adsorbs ambient hydrocarbons with time. A freshly prepared SLA surface is close to fully wetting, and the frequently quoted values above 130° describe an aged, hydrocarbon-contaminated surface at an unspecified storage interval rather than SLA as such. We therefore describe conventional SLA as losing hydrophilicity on storage rather than assigning it a fixed contact angle.^17^ Current studies have shifted focus toward nanoscale surface engineering, bioactive coatings, and “smart” stimuli-responsive systems, including titanium dioxide (TiO_2_) nanotubes, laser-induced periodic surface structures, metal–organic frameworks, and on-demand therapeutic release platforms (Figure 1).^18,19^

**Figure 1.**
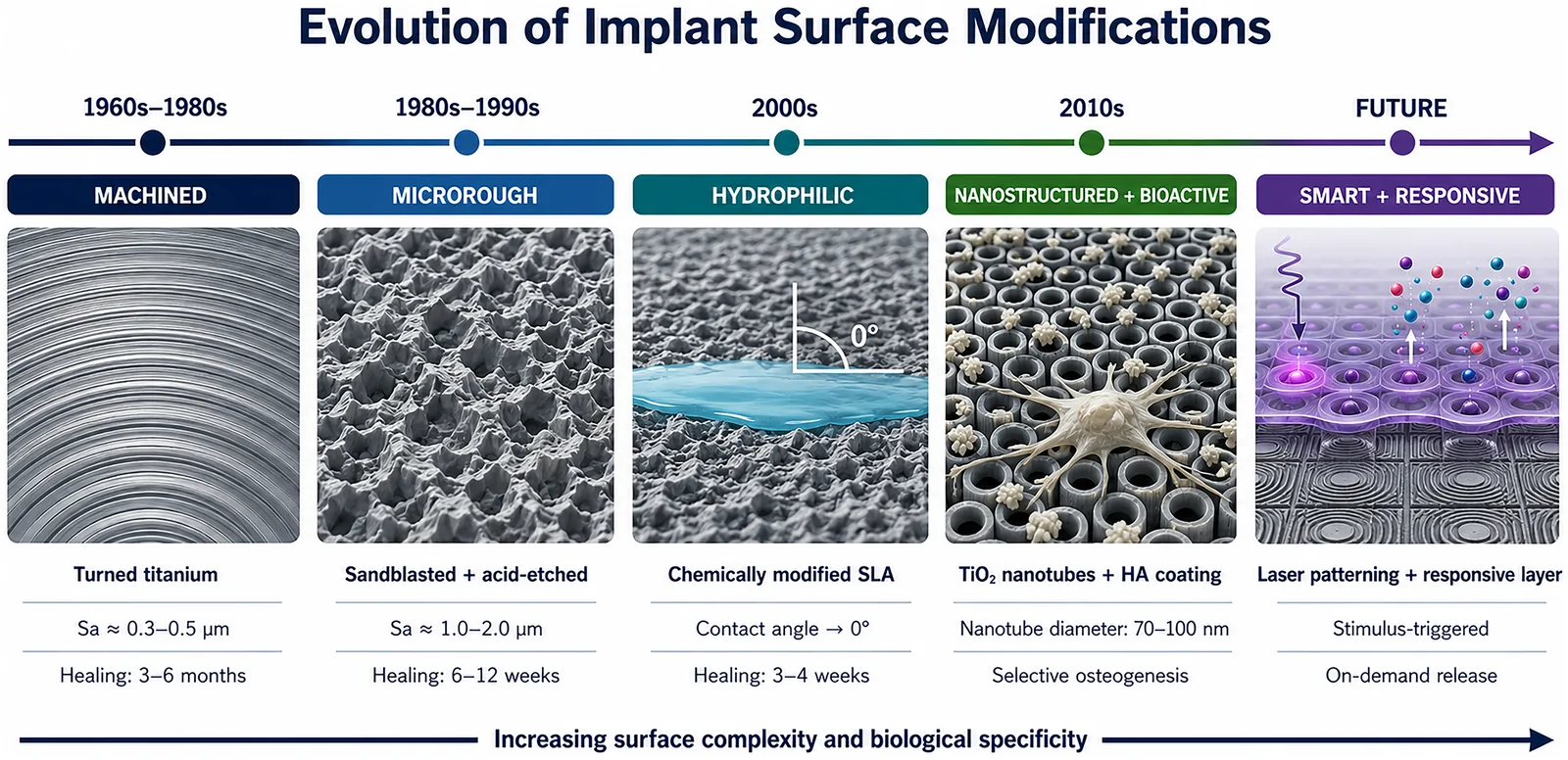
Evolution of implant surface modifications: from machined to bioactive and smart surfaces. Schematic timeline of dental implant surface development in five stages, with the approximate roughness domain and healing interval indicated beneath each panel. Machined (1960s to 1980s): turned commercially pure titanium showing uniform concentric machining grooves across the whole field, Sa approximately 0.3–0.5 micrometers, with a healing interval of 3–6 months. Microrough (1980s to 1990s): SLA titanium with irregular pits at the cell scale, Sa approximately 1.0–2.0 micrometers, with a healing interval of 6–12 weeks. Hydrophilic (2000s): chemically modified SLA held in protective storage, shown with a fully wetting droplet and a water contact angle approaching 0 degrees, with a healing interval of 3–4 weeks. Nanostructured and bioactive (2010s): ordered TiO_2_ nanotube arrays 70–100 nm in diameter together with hydroxyapatite coatings, supporting selective osteogenic differentiation. Smart and responsive (future): nanoscale laser patterning combined with stimuli-responsive layers permitting stimulus-triggered, on-demand release. Surface complexity and biological specificity increase from left to right. The panels are schematic representations; they are original artwork prepared by the authors and are not experimental micrographs.

SLA: sandblasted and acid-etched; TiO_2_: titanium dioxide; Sa: arithmetical mean height.

This narrative review examines next-generation dental implant surface modifications designed to enhance osseointegration, encompassing nanoengineered architectures, bioactive and antimicrobial coatings, stimuli-responsive drug delivery systems, additive manufacturing, advanced surface-processing technologies, and emerging materials such as graphene oxide and polyetheretherketone (PEEK). We integrate evidence from in vitro, in vivo, and clinical studies to identify key translational gaps and define research priorities, including artificial intelligence (AI)-guided surface design, with the goal of facilitating the translation of these technologies into routine clinical practice.

### Scope, aim, and search strategy

This article is a narrative review rather than a systematic review or meta-analysis; therefore, no International Prospective Register of Systematic Reviews (PROSPERO) registration was undertaken, and the Preferred Reporting Items for Systematic Reviews and Meta-Analyses (PRISMA), Consolidated Standards of Reporting Trials (CONSORT), Strengthening the Reporting of Observational Studies in Epidemiology (STROBE), and Animal Research: Reporting of In Vivo Experiments (ARRIVE) reporting guidelines do not apply. The review was guided by the Scale for the Assessment of Narrative Review Articles (SANRA),^20^ and each SANRA item—justification of importance, statement of aims, description of the literature search, referencing, scientific reasoning, and appropriate presentation of data—was addressed during preparation. The aim was to synthesize preclinical and clinical evidence on next-generation dental implant surface modifications, identify where the evidence does and does not support clinical translation, and define research priorities. PubMed/MEDLINE, Scopus, Web of Science Core Collection, and Embase were searched from database inception through 30 June 2026, with an emphasis on literature published from 2015 onward. This search was supplemented by hand-searching the reference lists of retrieved articles and relevant reviews. The search strategy combined controlled vocabulary and free-text terms across three concept blocks: (a) “dental implant” OR “titanium implant” OR “osseointegration” OR “bone–implant interface”; (b) “surface modification” OR “surface topography” OR “nanotube” OR “nanostructure” OR “bioactive coating” OR “antimicrobial coating” OR “stimuli-responsive” OR “additive manufacturing” OR “plasma treatment” OR “laser texturing” OR “atomic layer deposition”; and (c) “peri-implantitis” OR “osteoimmunomodulation” OR “macrophage polarization.” Eligible sources included peer-reviewed in vitro, in vivo, and clinical studies as well as systematic reviews and meta-analyses published in English that reported surface characterization together with a biological or clinical outcome. Conference abstracts, nonpeer-reviewed preprints, case reports involving fewer than five implants, and articles without accessible full text were excluded. Landmark historical papers were retained irrespective of publication date. Two authors independently screened records at the title and abstract level and subsequently at the full-text level, with disagreements resolved through discussions. Because the synthesis was narrative, no formal risk-of-bias scoring or quantitative pooling was performed. No preprint of this manuscript has been posted to a preprint server, and the manuscript has not been published or submitted elsewhere.

## Fundamentals of osseointegration

### Molecular and cellular mechanisms

Osseointegration proceeds through a precisely orchestrated sequence of cellular and molecular events at the bone–implant interface. Within seconds of implant placement, plasma proteins, including fibrinogen, fibronectin, and vitronectin, adsorb onto the surface, with adsorption kinetics and protein conformation dictated by surface chemistry, energy, and topography.^21^ These conditioned protein layers govern subsequent cellular recognition and adhesion, recruiting platelets, neutrophils, and macrophages that release cytokines and growth factors as well as mesenchymal stem cells (MSCs). MSCs differentiate along the osteogenic lineage through pathways regulated by Wnt/β-catenin and BMP signaling, culminating in osteoblast-mediated deposition of a mineralized matrix and formation of lamellar bone, thereby establishing durable mechanical anchorage (Figure 2).^12^

**Figure 2.**
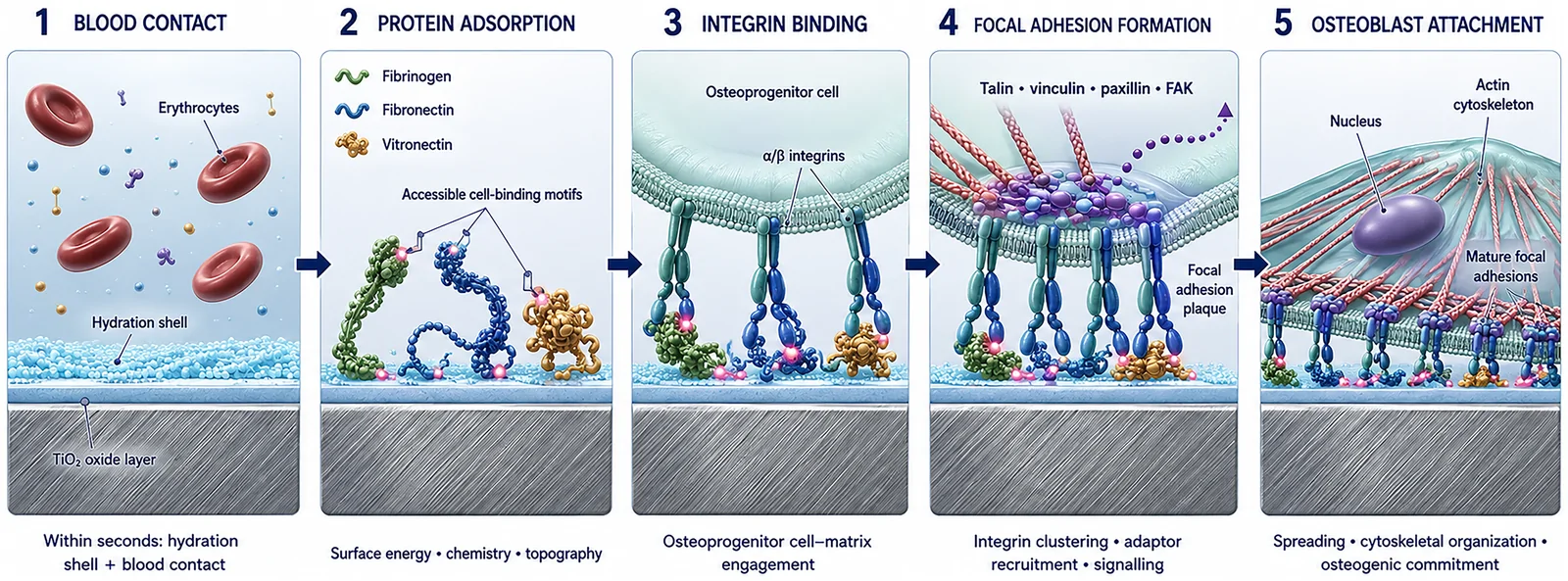
Cell–surface interaction cascade during early osseointegration. Sequence of events at the bone–implant interface following placement, shown from left to right, with the titanium substrate in cross-section beneath each panel. (a) *Blood contact*. Within seconds, a hydration shell forms, and erythrocytes and plasma reach the surface. (b) *Protein adsorption*. Fibrinogen, fibronectin, and vitronectin adsorb onto the oxide layer, and the conformation of the adsorbed layer, determined by surface energy, chemistry, and topography, governs which cell-binding motifs remain accessible. (c) *Integrin binding*. Osteoprogenitor cells engage the adsorbed protein layer through integrin receptors. (d) *Focal adhesion formation*. Integrin clustering recruits adaptor proteins into an adhesion plaque and initiates signal transduction. (e) *Osteoblast attachment*. Mature focal adhesions support cell spreading, cytoskeletal organization, and the onset of osteogenic differentiation. Original figure prepared by the authors; schematic representation, not experimental data.

### Surface properties and cellular behavior

Surface properties profoundly shape cellular behavior at the bone–implant interface, with topography, wettability, and chemistry acting in concert. Surface roughness influences cell adhesion, proliferation, and differentiation, with moderately rough surfaces typically optimizing osteoblast attachment and osteogenic differentiation.^22^ Hydrophilic surfaces with low water contact angles accelerate protein adsorption and cellular attachment relative to hydrocarbon-contaminated counterparts. The SLActive surface, stored to retain near-zero contact angles, has been associated with healing intervals of approximately 3–4 weeks compared with 6–12 weeks for conventional SLA. Reported contact angles for conventional SLA should be interpreted as time-dependent measurements rather than intrinsic constants, as wettability declines during storage as ambient hydrocarbons adsorb onto the surface; comparisons are meaningful only when the aging interval is specified. Beyond microscale features, nanoscale topography activates mechanotransduction pathways through focal adhesion remodeling and yes-associated protein and transcriptional coactivator with PDZ-binding motif (YAP/TAZ) signaling, whereas surface chemistry, particularly the presence of TiO_2_ versus contaminant carbon, strongly influences integrin engagement and downstream signaling.

### Osteoimmunology and macrophage polarization

The host immune response represents a critical yet historically underappreciated determinant of osseointegration outcomes. Macrophages exhibit functional plasticity, polarizing along a spectrum from pro-inflammatory M1 to anti-inflammatory and pro-healing M2 phenotypes; the timing and balance of this polarization profoundly influence subsequent bone formation.^23^ M1 macrophages predominate during the early postimplantation period and clear cellular debris and pathogens, whereas a timely transition toward M2 macrophages promotes the secretion of anti-inflammatory cytokines, including interleukin-10 (IL-10) and transforming growth factor beta 1 (TGF-β1), which support tissue repair, angiogenesis, and osteogenesis. Surface modifications can be engineered to influence macrophage polarization; hydrophilic surfaces, specific topographical features, and bioactive coatings that release immunomodulatory factors can enhance M2 polarization and accelerate osseointegration. This osteoimmunomodulatory paradigm—designing surfaces that actively modulate the immune response rather than simply minimizing inflammation—is reshaping next-generation surface design (Figure 3).

**Figure 3.**
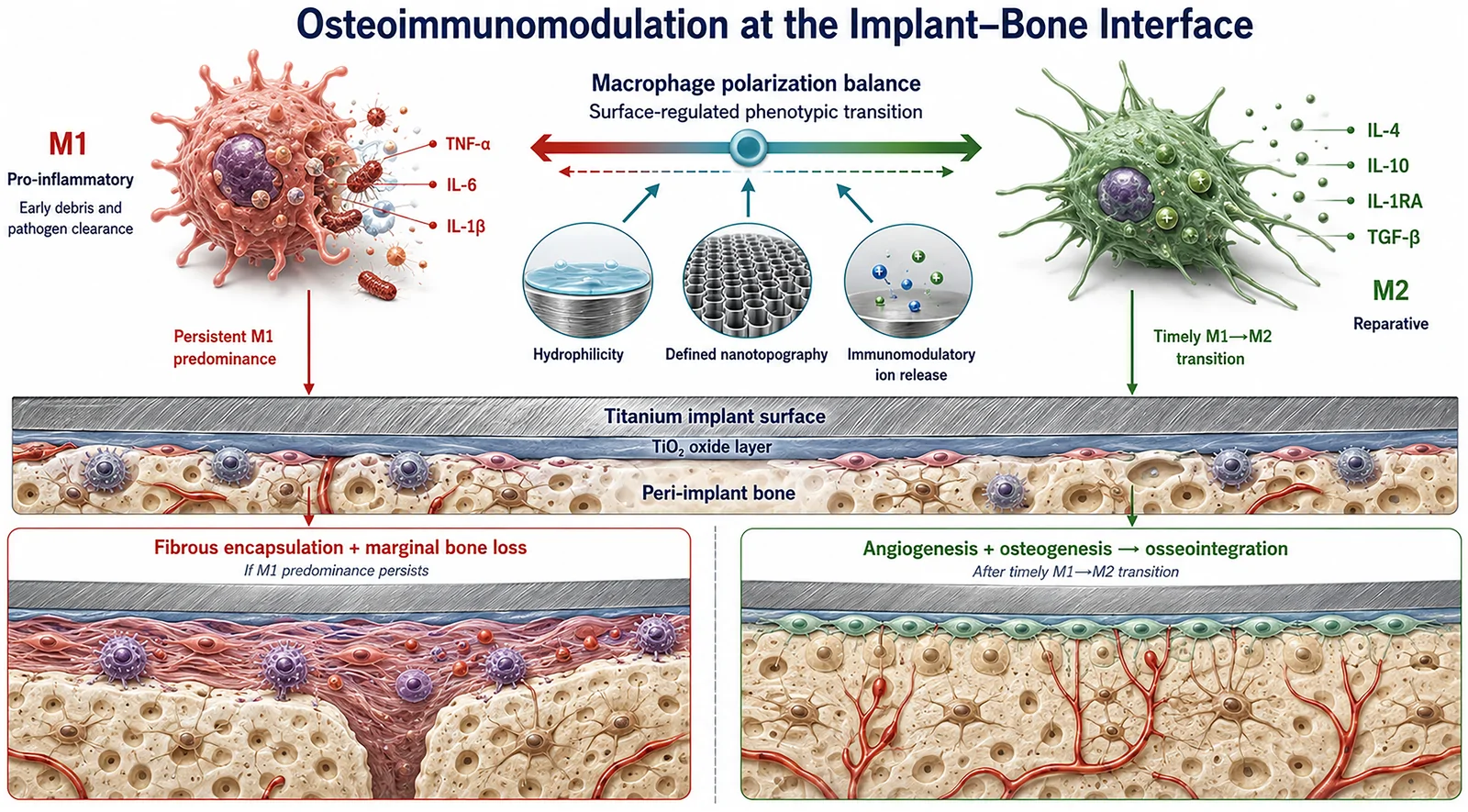
Osteoimmunomodulation at the implant–bone interface: M1/M2 macrophage polarization. Schematic of macrophage polarization at the implant–bone interface. Left: pro-inflammatory M1 macrophages predominate early and secrete TNF-α, IL-6, and IL-1β, clearing debris and pathogens. Right: the reparative M2 phenotype secretes IL-4, IL-10, IL-1RA, and TGF-β. Center: the balance between the two phenotypes is set by surface cues, of which hydrophilicity, defined nanotopography, and immunomodulatory ion release are indicated. The consequences at the titanium surface and the adjacent peri-implant bone are shown below. Persistent M1 predominance favors fibrous encapsulation and marginal bone loss, whereas timely transition from M1 to M2 supports angiogenesis and osteogenesis, leading to osseointegration. Original figure prepared by the authors; schematic representation, not experimental data.

TNF-α: tumor necrosis factor-alpha; IL: interleukin; IL-1RA: interleukin-1 receptor antagonist; TGF-β: transforming growth factor beta.

### Compromised conditions

Systemic conditions that compromise bone quality present significant clinical challenges. Diabetes mellitus can impair osseointegration through hyperglycemia-induced accumulation of advanced glycation end products, oxidative stress, and microvascular dysfunction; meta-analyses report higher early failure rates in patients with diabetes, although adequate glycemic control narrows this gap.^24^ Osteoporosis reduces bone density and quality, particularly in the maxilla, and conventional bisphosphonate therapy carries a small but recognized risk of medication-related osteonecrosis. Cigarette smoking impairs vascularization and wound healing, with implant failure rates approximately twice those in nonsmokers. These compromised states motivate the development of surface modifications that enhance osteogenesis and angiogenesis under suboptimal biological conditions, including bioactive coatings that release strontium, magnesium, or growth factors, to restore bone-forming capacity in at-risk populations.

## Nanoengineered surfaces

The emergence of nanoengineering has transformed implant surface design by enabling control of surface architecture at the nanometer scale, where it directly interfaces with cellular sensing machinery. Nanoscale features influence integrin clustering, focal adhesion maturation, and downstream signaling cascades that determine cell fate decisions.^25^ Engineered nanotopographies, including pillars, pits, gratings, and tubes, provide reproducible control over feature size, spacing, and chemistry, enabling correlations between specific architectural parameters and cellular responses and thereby accelerating rational surface design.

### TiO_2_ nanotubes: Fabrication, optimization, and biological response

TiO_2_ nanotube arrays (TiO_2_ NTAs) represent a paradigm shift in implant surface modification, offering precise control over nanoscale topography, surface area, and biological responses. Fabricated by electrochemical anodization in fluoride-containing electrolytes, these vertically oriented arrays exhibit tube diameters ranging from 15 to 200 nm and enable directly tunable biological effects.^26^ Diameter-dependent cellular responses are well established. Tubes with a diameter ranging from 15 to 30 nm promote osteoblast adhesion and proliferation, whereas those with a diameter ranging from 70 to 100 nm induce approximately 10-fold elongation of MSCs and selective osteogenic differentiation. Larger diameters, >100 nm, may impair cell adhesion because of excessive feature spacing. In vivo, anodized TiO_2_ nanotube surfaces provide up to a ninefold enhancement in bone bonding compared with smooth controls, supporting their translational potential for next-generation osseointegrating implants (Figure 4).

**Figure 4.**
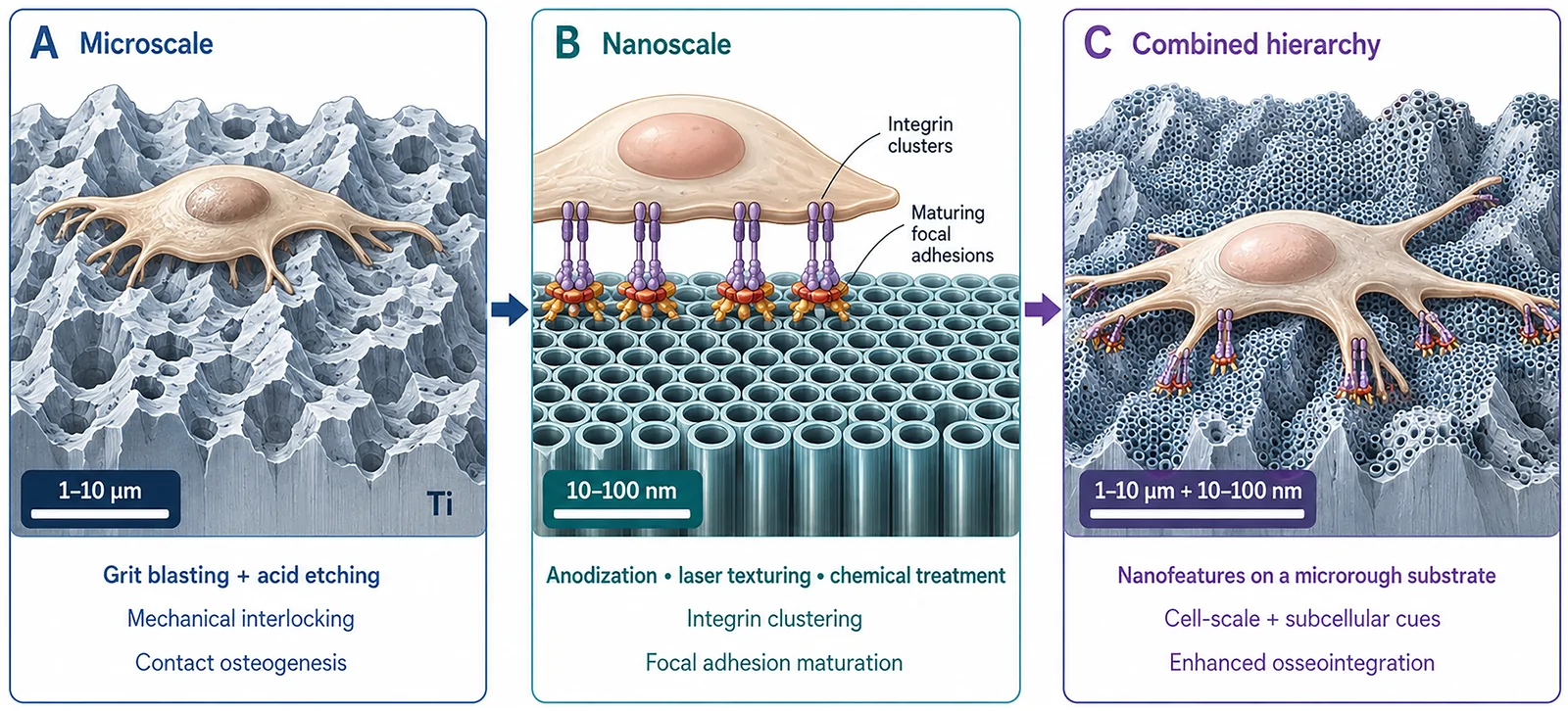
Hierarchical micro–nano surface architectures for enhanced osseointegration. Three panels with indicative scale bars. Left, microscale: features of approximately 1–10 micrometers generated by grit blasting and acid etching provide mechanical interlocking and promote contact osteogenesis. Center, nanoscale: ordered features of approximately 10–100 nm generated by anodization, laser texturing, or chemical treatment engage integrin clustering and focal adhesion maturation. Right, combined hierarchy: nanofeatures superimposed on a microrough substrate deliver cues at both the cell and the sub-cell length scales. The panels are schematic representations; they are original artwork prepared by the authors and are not experimental micrographs.

### Anodization and electrochemical treatments

Electrochemical anodization is the most widely employed technique for fabricating TiO_2_ NTAs, offering precise control over nanotube dimensions through manipulation of processing parameters.^27^ The technique uses a two-electrode electrochemical cell, with titanium as the anode and typically platinum or graphite as the cathode, immersed in a fluoride-containing electrolyte and operated under an applied direct current (DC) voltage. The formation mechanism proceeds through three distinct steps: field-assisted oxidation, which forms a compact TiO_2_ layer; field-assisted dissolution, in which Ti^4+^ cations migrate to the oxide/electrolyte interface; and chemical dissolution (etching) by fluoride ions, which creates the characteristic tubular morphology (Figure 5).^28^

**Figure 5.**
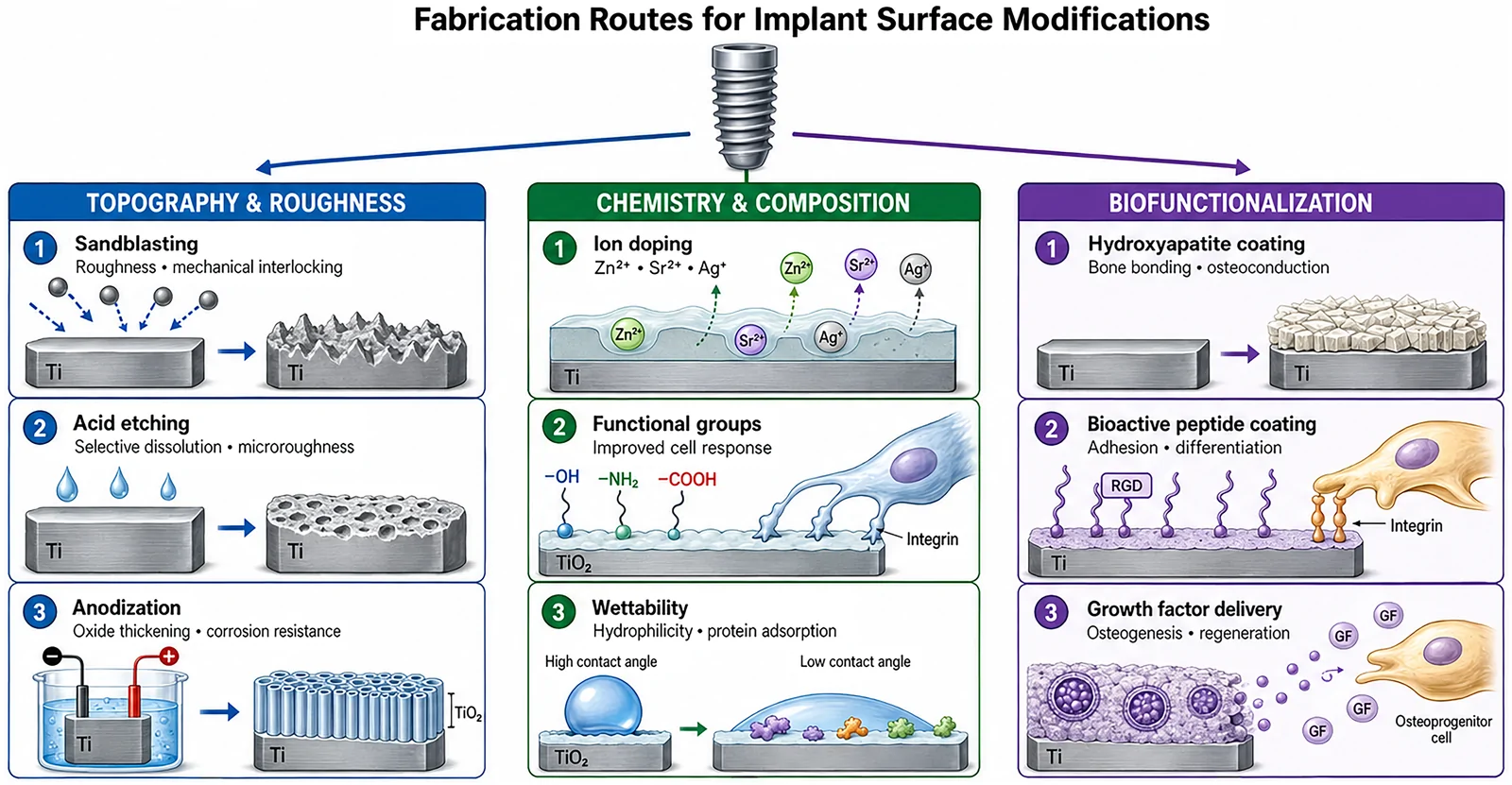
Fabrication routes for implant surface modifications: physical, chemical, and bifunctional approaches. Fabrication routes grouped by the surface property they alter. Routes that alter topography and roughness: sandblasting, which increases roughness and mechanical interlocking; acid etching, which creates microroughness by selective dissolution; and anodization, which thickens the oxide layer and improves corrosion resistance. Routes that alter surface chemistry and composition: ion doping, which incorporates bioactive ions such as zinc, strontium, and silver; introduction of functional groups, which improves cell response; and modification of wettability, which raises hydrophilicity and protein adsorption. Routes that add bioactive molecules: hydroxyapatite coating, which promotes bone bonding and osteoconduction; bioactive peptide coating, which enhances adhesion and differentiation; and growth factor delivery, which stimulates osteogenesis and regeneration. All three groups converge on bone formation and osseointegration. Acid etching and anodization are chemical and electrochemical processes, respectively, and are grouped with the topographical routes because their principal effect on the implant is a change in surface texture. Original figure prepared by the authors; schematic representation, not experimental data.

The evolution of anodization electrolytes has progressed through multiple generations: aqueous fluoride systems, which yield short, disordered nanotubes; viscous organic electrolytes (ethylene glycol with ammonium fluoride), which produce well-ordered, longer arrays exceeding 1 mm; and contemporary mixed-electrolyte systems incorporating ionic species or therapeutic ions that simultaneously dope the tube walls.^29^ Doping with strontium, calcium, magnesium, zinc, copper, or silver imparts antimicrobial, osteogenic, or angiogenic properties to nanotube surfaces, enabling multifunctional surfaces through a single processing step.

### Nanoscale topography and cell guidance

Nanoscale surface topography profoundly influences cellular behavior through mechanotransduction, with nanofeature dimensions ranging from 10 to 200 nm modulating cell adhesion, cytoskeletal organization, and gene expression. Cells sense nanotopography through integrin clustering and focal adhesion maturation, transducing mechanical cues into biochemical signals via RhoA/ROCK and YAP/TAZ pathways.^25^ Anisotropic nanopatterned gratings and aligned grooves direct cell elongation and orientation, whereas isotropic features influence cell spreading and differentiation independently of directional cues (Figure 6).

**Figure 6.**
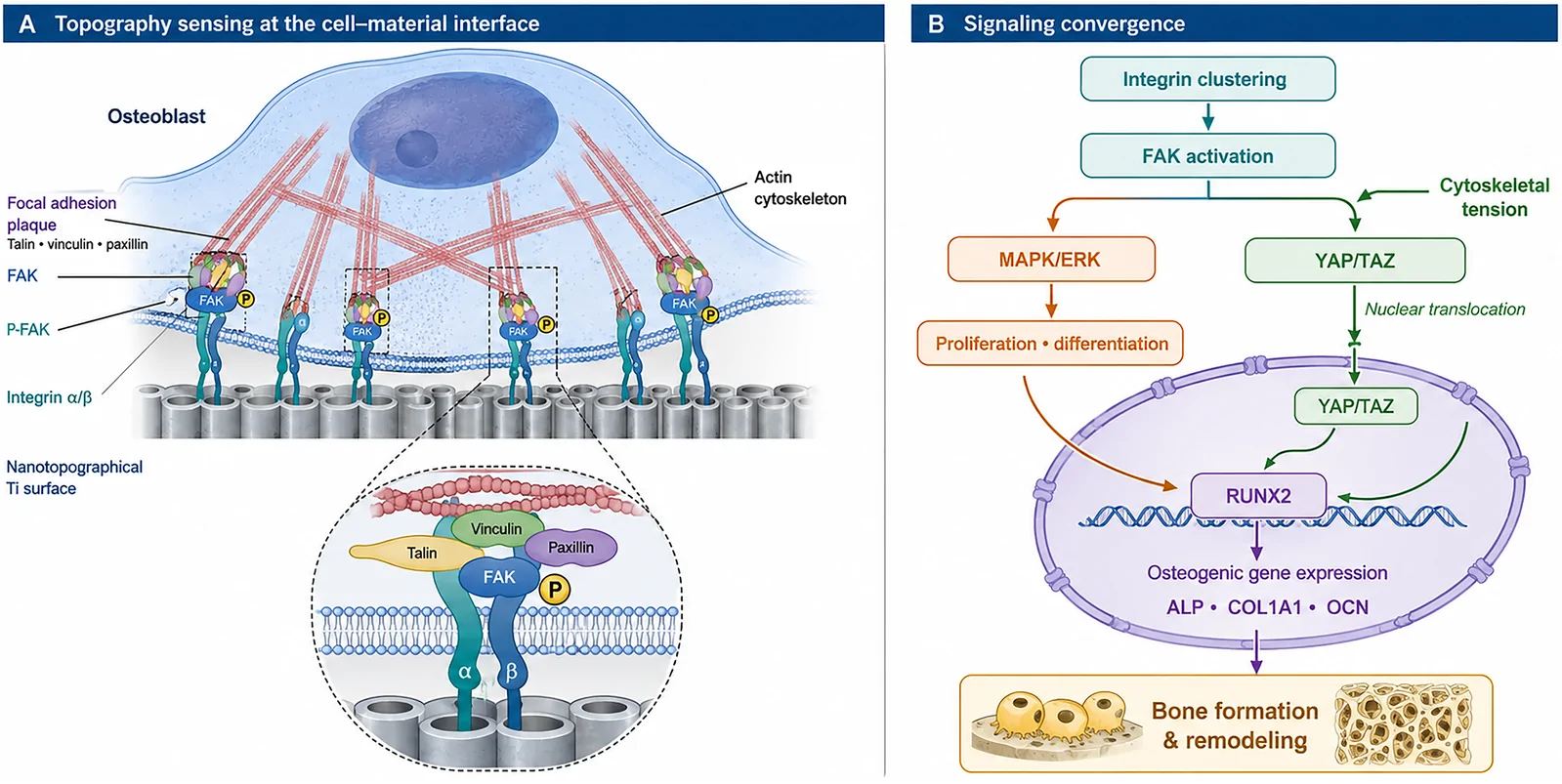
Mechanotransduction pathways activated by surface topography. Left: an osteoblast on a nanotopographical titanium surface, showing integrin engagement and assembly of a focal adhesion plaque. Right: the resulting signaling cascade. Integrin clustering activates focal adhesion kinase (FAK); signal transduction proceeds through the mitogen-activated protein kinase (MAPK) pathway, which regulates proliferation and differentiation, and through nuclear translocation of YAP/TAZ in response to mechanical cues; both converge on RUNX2-dependent osteogenic gene expression, driving bone formation and remodeling. Original figure prepared by the authors; schematic representation, not experimental data.

YAP/TAZ: yes-associated protein and transcriptional coactivator with PDZ-binding motif.

### Surface chemistry modifications at the nanoscale

Although topography provides physical cues, nanoscale surface chemistry modifications enable precise control of the biochemical interface between the implant and the host. Self-assembled monolayers (SAMs) of phosphonates, silanes, or thiols enable precise tailoring of surface functional groups. Bioactive peptide immobilization, including arginine-glycine-aspartic acid (RGD), lysine-arginine-serine-arginine (KRSR), and bone morphogenetic protein-2 (BMP-2)–derived sequences, directly elicits specific cellular responses, with KRSR and RGD originally designed and validated as osteoblast-adhesive sequences for chemical modification of biomaterials.^30^ These approaches were subsequently confirmed to improve osseointegration outcomes in vivo in a systematic review and meta-analysis of biomolecule-functionalized titanium surfaces.^31^ Click chemistry approaches enable orthogonal multifunctionalization, whereas plasma polymerization deposits ultrathin reactive layers that are compatible with diverse substrates and amenable to additional biomolecule attachment.

## Bioactive coatings

Bioactive coatings elicit biological responses through chemical cues that mimic those of native bone. These coatings include calcium phosphates, bioactive glasses, growth factor–immobilized surfaces, and natural polymer composites, each engineered to recapitulate aspects of the bone extracellular matrix (ECM) and accelerate osseointegration.^32^ It should be emphasized that this does not render the preceding topographical strategies passive. Microrough and nanostructured titanium surfaces, such as SLA, DAE, and TiO_2_ nanotube arrays, actively direct cell behavior through physical stimuli, namely topography and the mechanotransduction it evokes. In contrast, bioactive coatings direct cell behavior through biochemical stimuli. Both classes are active in terms of cellular stimulation; they differ in the modality of the cue rather than in whether a cue is delivered at all.

### Calcium phosphate (CaP) and hydroxyapatite (HA) coatings

CaP coatings, particularly HA (Ca_10_(PO_4_)_6_(OH)_2_), remain the most extensively studied bioactive coating materials because of their chemical similarity to the mineral phase of natural bone. A systematic review confirmed that HA surface treatment is effective in promoting osseointegration of titanium dental implants, with 12 of 15 included studies reporting favorable outcomes.^33^ The bioactivity of HA coatings derives from their capacity to release calcium and phosphate ions, which can be incorporated into the surrounding newly formed bone, stimulate alkaline phosphatase expression, and promote mineralization.

Nanostructured HA coatings have demonstrated promise, with in vivo studies in sheep models showing significantly higher bone-to-implant contact and bone area fraction occupancy compared with DAE surfaces at both 14 and 28 days postimplantation.^34^ Long-term clinical data confirm excellent durability: histomorphometric analysis of HA-coated implants that remained functional for approximately 11 years showed an average bone–implant contact of 75%, with 52%–70% residual HA coating coverage.^35^ Multiple deposition techniques have been developed, including plasma spraying, biomimetic wet chemical deposition under physiological conditions, and electrophoretic deposition, offering precise control over coating thickness. Recent studies have achieved needle-like nanostructured HA coatings at pH 6 that demonstrate 92.9% corrosion protection efficiency and superior biomineralization. Ion-substituted HA coatings incorporating zinc, strontium, magnesium, or silicon have emerged as next-generation formulations that provide enhanced osteoinduction, antibacterial activity, and controlled degradation.

### Bioactive glass (BG) compositions

BGs, first developed by Larry Hench in 1969, comprise silicon dioxide (SiO_2_)-based ceramics that release ionic dissolution products that stimulate osteoblast proliferation, differentiation, and angiogenesis.^36^ The classic 45S5 Bioglass formulation forms a carbonated HA layer in physiological fluids that bonds chemically to bone within hours. Contemporary mesoporous bioactive glass (MBG) coatings provide a high specific surface area for therapeutic ion or drug loading and tunable degradation kinetics. Doping with niobium enhances osteogenic and angiogenic activity through the Wnt/β-catenin and HIF-1α pathways; cerium provides antioxidant and antimicrobial functions; and zinc and copper confer antibacterial activity and stimulate bone formation. Strontium-doped BGs uncouple bone resorption from formation by modulating both osteoblast and osteoclast activity, which is particularly relevant to patients with osteoporosis, in whom bone homeostasis is dysregulated.

### Growth factor immobilization strategies

Growth factor delivery represents a biomimetic approach to enhancing osseointegration through the controlled presentation of osteoinductive proteins. BMP-2 is the most extensively studied osteoinductive factor for growth factor–functionalized implants. Bolus delivery has been associated with adverse effects, such as heterotopic ossification and inflammation, driving the development of strategies that immobilize BMP-2 on implant surfaces for sustained, localized release.^37^ Approaches include physical adsorption to nanoporous surfaces, covalent attachment via crosslinkers, encapsulation in degradable polymers, and tethering to heparin-functionalized surfaces that mimic native ECM presentation. Vascular endothelial growth factor (VEGF) co-delivery enhances angiogenesis and improves osseointegration in compromised bone, whereas platelet-derived growth factor (PDGF) accelerates early-phase healing. Sequential or spatially patterned multifactor delivery, combining angiogenic and osteogenic factors with distinct release kinetics, better recapitulates the natural bone-healing cascade than single-factor approaches and offers a path toward biomimetic surfaces with meaningful clinical efficacy.

## Antimicrobial surface modifications

The term implant-associated infection conflates two distinct clinical entities, and this section separates them. The first is true infection of the implant surface, that is, bacterial colonization of the fixture during or shortly after placement, which is the principal cause of early implant failure before osseointegration is established. The second is peri-implantitis, in which microbial colonization of the transmucosal interface is not itself the disease but the persistent stimulus that precedes and provokes the host response responsible for progressive inflammatory destruction of the peri-implant tissues and marginal bone.^10^ The two conditions require different surface solutions: the former is addressed by perioperative and early-phase antimicrobial activity of finite duration, whereas the latter is addressed by durable, nonfouling, immunomodulatory surface behavior in the transmucosal region. Aggregate peri-implantitis prevalence is 19.53% of patients and 12.53% of implants.^6^ In the sections that follow, antimicrobial denotes demonstrated activity over the stated observation period, not an implied guarantee of lifelong surface sterility. Surface modifications that combine antimicrobial activity with maintained or enhanced osteogenic properties have therefore become a major focus of contemporary research, with strategies ranging from contact-killing surfaces and antimicrobial-eluting coatings to immobilized peptides and light-activated approaches.^38^ A necessary caveat applies to all release-based systems described below: a dental implant is expected to function for decades, whereas the drug or ion payload that a surface coating can carry is finite. Thus, elution strategies are best understood as protecting the infection-vulnerable perioperative and early-healing window rather than as providing lifelong protection. Contact-killing and nonfouling chemistries avoid depletion in principle, but they are vulnerable to fouling by the acquired pellicle and to mechanical and chemical wear, and evidence for retained efficacy at clinically relevant time scales is currently lacking for every strategy discussed.

### Silver and other metal nanoparticle systems

Silver nanoparticles (AgNPs) are the most extensively studied antimicrobial nanomaterials for implant applications, exhibiting broad-spectrum activity against Gram-positive and Gram-negative bacteria, including methicillin-resistant *Staphylococcus aureus* and antibiotic-resistant strains.^39^ Their multimodal mechanisms of action, including membrane disruption, intracellular reactive oxygen species (ROS) generation, DNA damage, and interference with respiratory enzymes, may help minimize the development of resistance. Particle size critically modulates both antimicrobial efficacy and cytotoxicity: 10–20 nm AgNPs typically maximize antibacterial activity while preserving acceptable biocompatibility. Incorporation strategies include direct embedding in titanium oxide layers via plasma electrolytic oxidation, sputter deposition, electrochemical reduction within nanotube arrays, and polymer–AgNP nanocomposite coatings. Beyond silver, copper nanoparticles exhibit both antimicrobial and angiogenic activity by activating the HIF-1α pathway, whereas zinc oxide nanoparticles combine antimicrobial activity with osteogenic stimulation. The principal challenge remains achieving sustained antimicrobial release without cumulative cytotoxicity to host tissues; current strategies include burst-and-sustained release profiles, targeted ion exchange, and stimuli-responsive systems that release active species only in the presence of bacterial triggers.

### Antimicrobial peptide (AMP) coatings

AMPs have emerged as promising alternatives to conventional antibiotics because of their broad activity, multiple mechanisms of action, and low propensity to elicit resistance.^40^ GL13K, a synthetic peptide derived from human parotid secretory protein, has been extensively studied for implant applications and demonstrates bactericidal activity against periodontal pathogens, including *Porphyromonas gingivalis*, *Aggregatibacter actinomycetemcomitans*, and *Fusobacterium nucleatum*, at micromolar concentrations. GL13K-coated titanium surfaces show a 99% reduction in bacterial biofilm formation while preserving osteoblast adhesion and proliferation. HHC-36, a synthetic cationic peptide, provides similarly broad activity, with covalently immobilized HHC-36 on titanium reducing *S. aureus* colonization by 95%–99% and enhancing osteogenic differentiation. Surface immobilization is achieved through covalent coupling, layer-by-layer assembly, or polymer-tethered linkers; orientation and surface density critically influence both antimicrobial activity and biocompatibility.^41^ Resistance evolution to AMPs proceeds significantly more slowly than with conventional antibiotics, representing a major translational advantage. Current research focuses on peptide design optimization, multipeptide combinations, and stimuli-responsive activation systems, with several AMP-based implant coatings advancing through preclinical and early clinical evaluation.

### Photodynamic and photothermal approaches

Light-activated antimicrobial strategies offer antibiotic-independent approaches for combating implant-associated infections, with the additional advantage of spatial and temporal control. Photodynamic therapy (PDT) employs photosensitizers, such as porphyrins, phthalocyanines, or methylene blue, which generate ROS upon visible-light irradiation, producing localized antimicrobial activity with minimal collateral tissue damage.^42^ PDT-functionalized implant surfaces demonstrate sustained antimicrobial activity through repeated light activation. Photothermal therapy (PTT) uses near-infrared (NIR)–absorbing nanomaterials, such as gold nanorods, graphene oxide, and molybdenum disulfide (MoS_2_) nanosheets, which convert light to heat (typically 50°C–60°C), thereby thermally inactivating bacteria and disrupting biofilms. Recent advances combine PDT and PTT in synergistic systems with low-power irradiation and integrate stimuli-responsive triggers (e.g. pH and infection markers) that activate antimicrobial action only in the presence of pathological conditions, minimizing host tissue exposure. The fundamental challenge in antimicrobial surface development is achieving robust antimicrobial activity while preserving biocompatibility and supporting osseointegration. Many antimicrobial agents, such as silver and copper ions, certain antibiotics, and quaternary ammonium compounds, exhibit dose-dependent cytotoxicity to host cells, particularly osteoblasts, narrowing the therapeutic window. Strategies to balance these competing requirements include controlled-release profiles that deliver high antimicrobial concentrations during the early infection-vulnerable period, followed by reduced release during osseointegration; selective antimicrobial mechanisms that target bacteria-specific structures while sparing host cells; bacteria-responsive release systems that activate only upon pathogen detection; and multilayered surfaces with separate antimicrobial and osteogenic functional layers.^43^ Standardized in vitro assays that combine bacterial and osteoblast coculture with long-term in vivo evaluation are essential for validating the safety and efficacy of next-generation antimicrobial surfaces before clinical translation.

## Smart and stimuli-responsive surfaces

Conventional antimicrobial coatings release therapeutic agents at predetermined rates regardless of clinical need, often resulting in inefficient drug use and accelerated cytotoxic effects. Stimuli-responsive surfaces address this limitation by releasing therapeutics only in response to pathological triggers, such as bacterial infection, inflammation, or biomaterial–host interactions, providing on-demand therapy that maximizes efficacy while minimizing exposure of healthy tissues. These intelligent platforms represent a fundamental shift toward responsive, microenvironment-aware surfaces. Two practical constraints on this entire class of surfaces are underinvestigated and should be stated plainly. 1. *Mechanical integrity during insertion*. Coatings and grafted layers on a threaded fixture are subjected to shear and abrasion as the implant is driven through cortical bone, yet delamination and third-body debris generation are rarely quantified. 2. *Terminal sterilization*. Gamma and electron-beam irradiation can induce chain scission, cross-linking, and oxidation in polymeric and peptidic coating components, whereas steam sterilization can hydrolyze linkers and denature immobilized proteins. Thus, the sterilized surface may not be the same as the surface that was originally characterized. Poststerilization characterization and insertion-torque-simulated wear testing should be considered minimum requirements for translational claims.

### pH-responsive drug delivery systems

Bacterial infections create acidic microenvironments through metabolic acid production, lowering the local pH from a physiological value of 7.4 to approximately 5.5–6.5. pH-responsive coatings exploit this gradient through polymer matrices, gating molecules, or switchable bonds that release antimicrobials only under acidic conditions. Layer-by-layer chitosan/alginate coatings preferentially degrade at low pH, releasing loaded antibiotics, whereas benzoic-imine and acid-labile hydrazone linkers cleave to liberate active payloads.^44^ These systems demonstrate excellent specificity, releasing 70%–90% of the payload at pH 5.5 versus less than 10% at pH 7.4, providing a high signal-to-noise ratio for infection-targeted therapy.

### Metal–organic frameworks (MOFs) as carrier platforms

MOFs have emerged as versatile carrier platforms for stimuli-responsive drug delivery in implant applications, offering exceptional surface areas exceeding 4000 m^2^/g, tunable pore architectures, and chemically diverse functionalization opportunities.^45^ Zeolitic imidazolate framework-8 (ZIF-8), a zinc-based zeolitic imidazolate framework, has received particular attention because pH-triggered degradation in acidic environments releases both encapsulated therapeutics and antimicrobial Zn^2+^ ions. Implant surfaces functionalized with ZIF-8 exhibit sustained antimicrobial release for more than 30 days while supporting osteogenic differentiation. MOF integration strategies include direct growth on implant surfaces via solvothermal synthesis, electrostatic deposition, and incorporation within polymer composite coatings.^46^ The controlled degradation of MOFs in physiological environments enables sustained therapeutic delivery while avoiding burst release.

### Enzyme-responsive coatings

Bacterial infections induce the overexpression of specific virulence factors, including hyaluronidases, lipases, and proteases, which serve as molecular triggers for enzyme-responsive drug-release systems. Hyaluronic acid-based coatings selectively degrade in the presence of bacterial hyaluronidases, releasing antimicrobial payloads only when pathogens are present.^47^ Lipase-cleavable phospholipid coatings undergo similar bacteria-triggered degradation, whereas protease-responsive polymeric matrices have been engineered with cleavage sequences specific to *Staphylococcus aureus* virulence factors. These enzyme-responsive systems achieve high specificity for bacterial infections while sparing host tissues. Their combination with pH-responsive triggers further refines selectivity, providing dual-stimuli activation that minimizes off-target effects and supports prolonged implant function.

## Additive manufacturing approaches

### Porous structure design and optimization

Lattice structures represent a paradigm shift from solid implants to architectures with controlled porosity, mechanical biomimicry, and enhanced osseointegration. Triply periodic minimal surfaces (TPMS), including gyroid, diamond, and Schwarz primitive geometries, provide mathematically defined, mechanically efficient porous architectures that mimic trabecular bone topology while maintaining structural integrity.^48^ Pore size critically determines biological response: 300–600 µm pores optimize bone ingrowth and vascularization, whereas smaller pores (100–300 µm) promote initial cell attachment. Strut thickness, surface area, and permeability further modulate cellular response and fluid transport. Functionally graded porosity transitions from solid cortical regions to highly porous cancellous regions, mimicking native bone architecture and reducing stress shielding. Computational design tools, finite element analysis, and topology optimization enable the creation of patient-specific geometries optimized for both mechanical and biological performance. Recent advances in additive manufacturing have achieved sufficient resolution to print micro- and nano-scale features within larger lattice structures, creating multiscale architectures with hierarchical organization analogous to natural bone, thereby accelerating both early integration and long-term remodeling.^49^

### Patient-specific implant customization

The integration of cone-beam computed tomography (CBCT), computer-aided design (CAD), and additive manufacturing has enabled patient-specific implant fabrication tailored to individual anatomy and mechanical requirements.^50^ Custom geometries optimize fit in challenging anatomical situations, including atrophic ridges, large defects, and cases requiring guided bone regeneration. Subperiosteal implants, historically limited by manufacturing constraints, have been revitalized through three-dimensional (3D) printing, offering reliable solutions for severely resorbed maxillae. Patient-specific surface texturing varies topography across implant regions, optimizing cortical anchorage while promoting cancellous integration. Cost-effectiveness improves as printing technologies mature, with patient-specific implants increasingly competitive with stock components for complex cases.

### Four dimensional (4D) printing and shape-memory materials

4D printing extends additive manufacturing capabilities by incorporating time as a fourth dimension: smart materials change shape, properties, or function in response to environmental stimuli, including temperature, pH, and mechanical load. Shape-memory nickel–titanium (NiTi) alloys, fabricated via selective laser melting, undergo programmable shape transformation that enables minimally invasive implant placement and postimplantation deployment to optimize fit.^51^ Heat-activated NiTi implants compress for insertion and expand at body temperature to engage surrounding bone, distributing load and reducing micromotion. Pseudoelastic NiTi behavior provides mechanical compliance that approaches that of cortical bone, thereby mitigating stress shielding. Beyond NiTi, magnesium-based alloys with tunable degradation, biodegradable polymers with shape memory, and stimuli-responsive hydrogel composites are under active investigation. Self-deploying scaffolds that expand to fill bony defects and dynamically adjust porosity in response to bone remodeling represent a particularly promising direction for guided bone regeneration. Although 4D printing for dental implants remains predominantly preclinical, integration with bioactive coatings and growth factor delivery offers a path toward truly responsive, biology-aware implant systems suited to dynamic clinical environments.

## Advanced processing technologies

### Femtosecond laser surface texturing

Femtosecond laser texturing has emerged as a breakthrough technology for implant surface modification, exploiting ultrashort pulses that ablate material with minimal heat transfer to surrounding tissue. This nonthermal processing produces precise surface features without altering the underlying microstructure or introducing thermal damage.^52^ Laser-induced periodic surface structures (LIPSS) generate spontaneously organized nanopatterns with subwavelength periodicity, whereas microscale grooves, pits, and pillars are achieved through scanning strategies. Hierarchical micro–nano architectures that combine microscale topography with nanoscale LIPSS provide multiscale cues for cellular responses. Surface chemistry can be tuned by the processing gas atmosphere: oxygen produces superhydrophilic surfaces ideal for bone integration, whereas nitrogen yields nitride coatings with enhanced wear resistance. Studies demonstrate enhanced osteoblast adhesion, accelerated osseointegration, and reduced bacterial colonization on femtosecond-laser-textured surfaces compared with conventional treatments.^53^ Clinical translation is advancing with several commercial systems incorporating femtosecond-laser texture, although throughput and cost remain limiting factors for widespread adoption.

### Plasma surface treatments

Plasma treatment has gained prominence as a versatile surface-modification approach that combines surface cleaning, activation, and chemical functionalization in a single processing step. Cold atmospheric plasma generates reactive species, radicals, ions, electrons, and ultraviolet (UV) photons that modify surface chemistry and topography without altering the bulk material. Argon plasma produces clean, hydrophilic surfaces with enhanced protein adsorption and cellular attachment; oxygen plasma creates oxidized surfaces with abundant hydroxyl groups for biomolecule conjugation; nitrogen and ammonia plasmas deposit nitrogen-containing functional groups that enhance osteoblast response.^54^ Plasma-deposited coatings and thin films deposited from precursor gases provide tailored surface chemistry with excellent adhesion. Bioactive plasma coatings incorporating calcium phosphate or therapeutic ions deliver controlled bioactivity.^55^ Plasma functionalization of polymers has been established practice since the 1980s and is not itself a recent advance; current interest lies rather in atmospheric-pressure and low-temperature systems that permit in-line, chamber-free treatment of assembled devices and of bioactive ceramic and composite substrates without thermal degradation.^55^

### Atomic layer deposition (ALD)

ALD enables precise control of implant coating thickness, composition, and uniformity at the atomic scale. The self-limiting surface reactions characteristic of ALD enable deposition of coatings with sub-nanometer-thick control on complex 3D geometries, including porous lattices and threaded implant surfaces. ALD-deposited TiO_2_ provides protective barriers preventing metal ion release while enhancing biocompatibility; HA-mimicking calcium phosphate coatings offer bioactive surfaces with controlled crystallinity and dissolution.^56^ Multilayered ALD architectures combine functional layers, including antimicrobial, osteogenic, and anticorrosion layers, within a single coating system. Therapeutic ion incorporation via ALD doping enables controlled release of antimicrobial silver, copper, or zinc ions or osteogenic strontium and magnesium ions. Although ALD remains constrained by relatively slow deposition rates and equipment costs, ongoing process development and scale-up are advancing the translation of ALD from research to commercial implant coating applications.

## Novel materials and emerging approaches

### Graphene and graphene oxide (GO) coatings

Graphene and GO have emerged as transformative coating materials for dental implants, offering unique combinations of mechanical strength, electrical conductivity, and biological activity. GO coatings enhance osteoblast proliferation and osteogenic differentiation through surface roughness effects, surface chemistry interactions, and modulation of cell signaling pathways.^57^ Mechanical reinforcement of polymer composites with GO improves wear resistance and fatigue performance, critical considerations for long-term implant function. Electrical conductivity enables electrically stimulated osteogenesis: applied electrical fields enhance osteoblast proliferation and matrix mineralization on GO-coated surfaces. Antimicrobial activity arises from membrane disruption by sharp graphene edges and oxidative stress, providing inherent resistance to bacterial colonization. Functionalization with growth factors, antimicrobial peptides, or therapeutic ions further enhances biological performance. Despite promising preclinical results, clinical translation faces challenges, including standardization of synthesis methods, long-term biocompatibility validation, and clarification of regulatory pathways for novel nanomaterials, all of which remain active areas of investigation.

### PEEK and polymer-based implants

PEEK has emerged as a formidable competitor to metallic implants, offering an elastic modulus closer to cortical bone (3–4 vs. 110 GPa for titanium), thereby reducing stress shielding, which is particularly important in load-bearing applications. PEEK exhibits excellent biocompatibility, chemical resistance, and radiolucency, which facilitate postoperative imaging. Inherent bioinertness, however, limits direct osseointegration, thereby motivating extensive research on surface modification. Approaches include plasma treatment to introduce polar groups, sulfonation to create surface roughness and exposed sulfonate groups, HA coating via plasma spray or sol-gel deposition, and incorporation of bioactive fillers such as BG or HA particles.^58^ Carbon-fiber-reinforced PEEK provides mechanical properties approaching those of titanium with retained low elastic modulus. Surface-modified PEEK demonstrates osseointegration approaching that of titanium implants in preclinical and early clinical studies. Persistent translational challenges include long-term mechanical performance, surface coating durability, and cost-effectiveness compared with established titanium alternatives, although clinical adoption is increasing in selected applications.

### Biodegradable metallic implants

Biodegradable metals represent a paradigm shift from permanent implants, offering temporary structural support followed by controlled degradation, thereby eliminating the need for removal surgery. Magnesium alloys provide mechanical properties similar to bone, biocompatible degradation products, and stimulation of bone formation through magnesium-ion-mediated osteogenesis. Iron-based and zinc-based alloys offer alternative degradation profiles with extended functional lifetimes.^59^ Degradation rate control remains the primary challenge: rapid degradation can lead to excessive hydrogen gas evolution and structural failure before adequate bone healing, whereas overly slow degradation can negate the principal advantage. Surface modifications, such as calcium phosphate coatings, polymer coatings, micro-arc oxidation, and rare earth element alloying, modulate degradation kinetics and enhance biocompatibility. Applications particularly suited to biodegradable metals include pediatric implants where growth accommodation is essential, bone fracture fixation devices, and craniomaxillofacial reconstruction with anticipated remodeling. Although still predominantly preclinical for dental applications, ongoing trials in orthopedic and cardiovascular settings demonstrate the translational potential of biodegradable metallic implants, with selected magnesium-based devices achieving regulatory approval in Europe and Asia.

### Zirconia-based ceramics

Zirconia (ZrO_2_) dental implants have emerged as a transformative alternative to titanium, particularly attractive for patients with metal sensitivities, those prioritizing aesthetic outcomes, and individuals seeking metal-free dentistry. Yttria-stabilized zirconia provides excellent biocompatibility, white tooth-like aesthetics, and reduced bacterial adhesion compared with titanium, supporting healthier peri-implant soft tissue environments. Surface modifications enhance osseointegration, which proceeds more slowly than with titanium in early studies. Sandblasting, acid etching, and laser texturing produce moderately rough surfaces; bioactive coatings, such as calcium phosphate, BG, and growth factor immobilization, actively promote bone integration.^60^ Recent meta-analyses suggest zirconia implant survival rates are comparable to titanium at 5–10-year follow-up, though longer-term comparative data remain limited. Mechanical concerns, particularly fracture resistance under cyclic loading, are being addressed through advanced manufacturing and design optimization. Clinical adoption has accelerated, with multiple commercial zirconia implant systems achieving regulatory approval and increasing use in aesthetically demanding cases.

## Clinical translation

Translating surface modifications from laboratory research to routine clinical practice remains the field's defining challenge. Although in vitro and preclinical studies demonstrate substantial gains in osseointegration, definitive evidence rests on clinical endpoints—implant survival, marginal bone stability, peri-implant tissue health, and patient-reported outcomes measured over extended follow-up periods. The sections below summarize contemporary clinical evidence, long-term survival data, regulatory frameworks, and health-economic considerations that govern adoption.

### Clinical trial evidence for surface modifications

Meta-analysis data indicate that bioactive surface modifications meaningfully improve osseointegration. A systematic review and meta-analysis of bioactive surface modifications reported an overall mean difference in bone-to-implant contact of 7.29 (95% CI: 2.94–11.65) in favor of surface-modified implants, with collagen-based coatings associated with accelerated early integration and BMP-2 combinations with the largest gains.^61^ This estimate derives predominantly from animal histomorphometry and should not be interpreted as a clinical outcome. SLA surfaces remain the most clinically validated; over 10 years in 511 SLA implants among 303 partially edentulous patients, Buser et al. reported a 97.0% success rate, 98.8% survival, and a 1.8% incidence of peri-implantitis.^13^ The hydrophilic SLActive modification preserves a near-zero water contact angle until placement, in contrast to the progressive loss of wettability seen in conventionally stored SLA, and has been associated with a shortening of the healing interval from approximately 6–12 weeks to approximately 3–4 weeks. A 10-year multicenter study of immediate and early loading with chemically modified SLA implants reported 97.6% cumulative survival, with 98.2% for immediate and 97.1% for early loading.^62^ A systematic review of 30 studies encompassing 1719 participants reported success rates of 90.9%–100% across contemporary surface modifications.^63^ It should be noted that these are heterogeneous observational data and that no adequately powered randomized trial has yet demonstrated superiority of any single contemporary surface over another for hard clinical endpoints.

### Long-term outcome data

Long-term outcome data provide the strongest foundation for clinical decision making. A systematic review with sensitivity meta-analysis of 18 studies of contemporary roughened solid-screw implants reported a summary 10-year implant-level survival of 96.4% (95% CI: 95.2%–97.5%), with a prediction interval of 91.5%–99.4%, although the authors treat the lower sensitivity estimate of 93.2% (95% CI: 90.1%–95.8%), which accounts for studies at higher risk of bias, as the more realistic figure.^64^ Extending the horizon, a 20-year meta-analysis reported a mean survival of 92% (95% CI: 82%–97%) in prospective cohorts, falling to 78% (95% CI: 74%–82%) after imputation for patients lost to follow-up.^65^ The gap between these two estimates is itself the most instructive finding, as it quantifies how much of the reportedlong-term success of implant therapy depends on assumptions about patients who were never re-examined. The largest single-center longitudinal cohort to date, comprising 10,871 implants in 4247 patients with follow-up extending to 22.2 years (mean, 4.5 ± 4.2 years), demonstrated cumulative implant-level survival of 98.9% at 3 years, 98.5% at 5 years, 96.8% at 10 years, and 94.0% at 15 years, with the Straumann tissue-level SLA implant comprising 55.2% of the cohort.^66^ These results confirm predictable long-term performance for surface-modified implants when combined with appropriate patient selection and maintenance. Despite favorable survival, peri-implant diseases continue to erode long-term outcomes. Aggregate peri-implantitis prevalence is 19.53% at the patient level (95% CI: 12.87–26.19) and 12.53% at the implant level;^6^ applying the case definitions of the 2017 World Workshop,^67^ those estimates are increased to 63.0% for peri-implant mucositis and 25.0% for peri-implantitis.^7^ Periodontitis history and cigarette smoking provide the most convincing evidence among peri-implantitis risk factors.^8^ The persistence of these complications reinforces the need for surfaces that couple durable control of the host inflammatory response at the transmucosal interface with enhanced osseous integration. Framing the problem solely as one of bacterial elimination is unlikely to be productive, as bacterial colonization of exposed surfaces cannot be prevented over a service life measured in decades, whereas the magnitude and resolution of the host response are, in principle, modifiable (Figure 7).^9^

**Figure 7.**
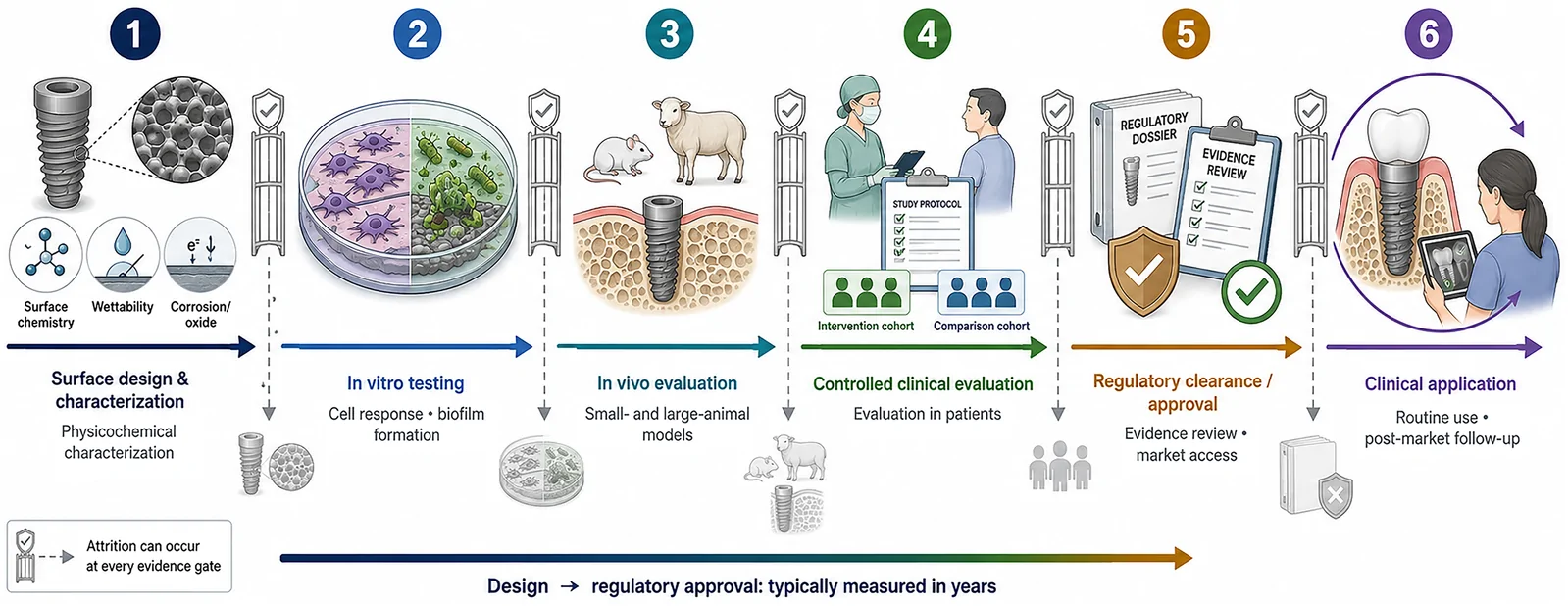
Bench-to-bedside translation pathway for surface-modified implants. The six stages through which a surface modification must pass before routine clinical use are as follows: (a) surface design and physicochemical characterization; (b) in vitro testing of cell response and biofilm formation; (c) in vivo evaluation in small- and large-animal models; (d) controlled clinical evaluation in patients; (e) regulatory clearance or approval; and (f) clinical application with postmarket follow-up. Attrition occurs at every transition, and the interval from initial design to regulatory approval is typically measured in years. Original figure prepared by the authors; schematic representation, not experimental data.

## Future perspectives

### AI-guided surface design

AI, advanced manufacturing, and smart biomaterials are converging to reshape implant dentistry. Machine learning models analyze large patient datasets, including medical history, diagnostic imaging, and longitudinal outcomes, to predict complications, recommend optimal designs, and support real-time surgical decision making. Current AI prognostic models report accuracies of 70%–96.13%, and robotic-assisted surgical systems already deliver placement precision that exceeds human capability, whereas deep-learning analysis of CBCT imaging automates anatomical segmentation, bone-quality assessment, and implant planning, reducing both procedure time and cost. These applications, however, concern clinical workflow rather than surface design, which is the subject of this review. Applied to surface design itself, machine learning is being used in four more specific ways. First, supervised regression and classification models trained on published surface-characterization datasets relate descriptors such as arithmetical mean height (Sa), developed interfacial area ratio (Sdr), skewness, nanotube diameter, and wettability to osteoblast adhesion, alkaline phosphatase activity, and bone-to-implant contact, enabling in silico ranking of candidate topographies before fabrication. Second, molecular dynamics and coarse-grained simulations of fibronectin, vitronectin, and albumin adsorption on titanium and TiO_2_ surfaces, coupled with machine-learned interatomic potentials, predict protein orientation and RGD-loop accessibility as a function of surface chemistry and curvature at a fraction of the cost of ab initio methods. Third, generative models, including variational autoencoders, generative adversarial networks, and diffusion models, are being used to propose nanotopographies conditioned on a desired cellular readout, an approach already demonstrated for topography-driven stem cell phenotype screening on large combinatorial micropattern libraries. Fourth, topology and shape optimization under multiobjective constraints, coupling finite element analysis of stress shielding with computational fluid dynamics of permeability, generates lattice and pore architectures that satisfy mechanical and mass-transport criteria simultaneously. Together, these approaches compress a combinatorial parameter space spanning composition, topography, chemistry, and therapeutic payload from thousands of candidates to a targeted set of high-probability designs suitable for focused experimental validation. The principal obstacle is not algorithmic but evidentiary: surface-characterization and biological outcome data are reported inconsistently across laboratories, and progress will depend on standardized, machine-readable reporting of surface parameters alongside biological endpoints (Figure 8).

**Figure 8.**
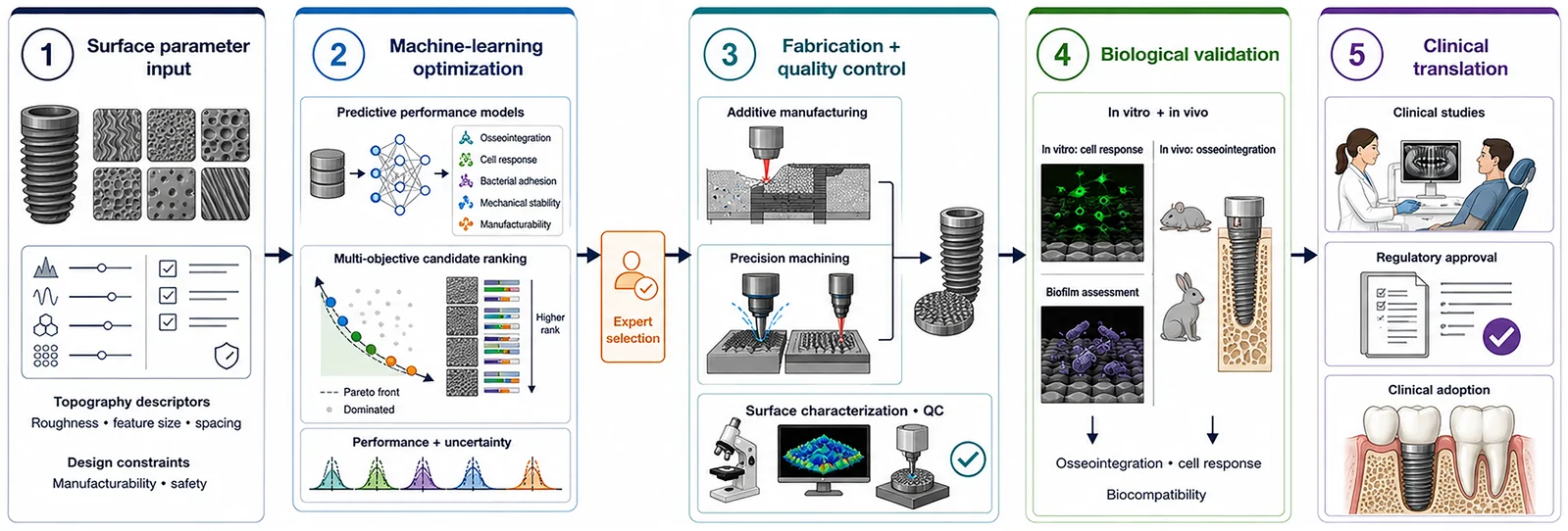
AI-guided implant surface design workflow. Proposed workflow for computationally guided surface design. (a) Surface parameter input: topography descriptors and design constraints are defined; (b) Machine learning optimization: predictive models estimate performance, and multiobjective search ranks candidate designs; (c) Fabrication: selected candidates are produced by additive manufacturing or precision machining, with surface characterization and quality control; (d) Biological validation: candidates undergo in vitro and in vivo evaluation of osseointegration, cell response, and biocompatibility; (e) Clinical translation: validated designs proceed to clinical studies, regulatory approval, and adoption. The dashed return path indicates experimental results being fed back into the training set, closing the design loop. Original figure prepared by the authors; schematic representation, not experimental data.

AI: artificial intelligence.

### Personalized surface modifications and theranostic implants

The future of surface modification is expected to shift toward personalized strategies informed by individual anatomy, medical history, genetic polymorphisms that affect bone metabolism and immune responses, and lifestyle risk factors. Patients with compromised bone quality may benefit from surfaces loaded with osteoinductive growth factors, whereas those at elevated risk of infection may benefit from enhanced antimicrobial functionality. Theranostic surfaces incorporating biosensors for pH, bacterial load, inflammatory markers, and mechanical strain could enable closed-loop systems that autonomously detect pathological shifts and trigger targeted therapeutic release.^68^ Embedded-sensor implants for continuous monitoring of osseointegration, occlusal forces, and peri-implant tissue health remain preclinical and lack standardization but represent a plausible trajectory. 4D printing pushes the envelope further: pH-responsive magnesium–zinc–calcium scaffolds fabricated via 4D bioprinting can dynamically adapt their pore architecture to match the phases of bone remodeling, thereby accelerating vascular ingrowth and osseointegration.^69^

### Immunomodulatory surface engineering and sustainability

Sequential immunomodulatory coatings that first establish anti-inflammatory M2 macrophage polarization and then promote angiogenesis and osteogenesis through the timed release of cytokines and growth factors more faithfully recapitulate the natural healing cascade than static modifications, with particular promise for patients with dysregulated immune responses.^70^ Parallel efforts in green-chemistry coating deposition, waste reduction in anodization and etching, and biodegradable coating components align with broader sustainability goals and may simultaneously reduce manufacturing costs and regulatory burden.

Significant challenges remain in translating laboratory innovations to clinical practice. Widespread adoption of AI and robotic technologies is constrained by high costs, steep learning curves, and the need for long-term validation across diverse populations. Regulatory pathways for multifunctional coatings remain complex; additive and 4D-printing approaches face limits in resolution, speed, and scalability; and embedded-sensor implants lack robust clinical validation and standardization. The opportunities, however, are substantial: peri-implant diseases affect approximately one in four implant patients, providing a clear target for antimicrobial and immunomodulatory surfaces; multifunctional architectures that integrate nanotopography, bioactive coatings, and controlled drug delivery offer pathways to address multiple failure modes through a single strategy; and biodegradable metallic implants that avoid secondary retrieval surgery hold particular promise in pediatric and sports-medicine applications.

### Relevance, novelty, and what this review adds

Several recent reviews have surveyed dental implant surface modification, and their emphases differ from ours in ways worth stating. Wennerberg and Albrektsson established the topographical framework that still governs the field,^22^ and Davies provided the histological account of contact osteogenesis on microrough surfaces on which much of the subsequent literature rests.^12^ More recent syntheses have concentrated either on titanium surface treatments for osteoblast response,^19^ on engineered surfaces for infection management,^11^ or on nanoengineering as a self-contained topic.^18^ This review differs in three respects. First, it places nanoengineered, bioactive, antimicrobial, stimuli-responsive, additively manufactured, and nontitanium surfaces within a single comparative frame rather than treating them as separate literatures, and Table 1 makes the level of evidence supporting each class explicit and comparable. Second, it treats osteoimmunomodulation as a design principle rather than an adjunct and applies that principle consistently, including to the framing of peri-implantitis and to the interpretation of what antimicrobial surface performance can and cannot deliver over a service life measured in decades. Third, it separates what has been demonstrated clinically from what has been demonstrated only in vitro or in animals and identifies the specific evidentiary and regulatory steps that stand between the two. The intended contribution is therefore a realistic map of the translational frontier rather than a catalog of laboratory achievements.

**Table 1.** Comparative analysis of next-generation implant surface-modification technologies.

Technology	Osseointegration enhancement	Antimicrobial effect	Key advantages	Clinical translation status
TiO_2_ nanotubes	Ninefold improvement in bone bonding (animal model)	Reduces *S. epidermidis* colonization	Tunable dimensions; drug delivery	Preclinical/Early clinical
Hydroxyapatite coatings	MD = 7.29 in BIC (meta-analysis of animal histomorphometry)	Limited intrinsic activity	Chemical similarity to bone	Commercially available
Femtosecond laser texturing	Superior to SLA surfaces	Photocatalytic antibacterial	Precise control; rapid processing	Preclinical validation
Graphene oxide	11.2-fold increase in OCN expression (in vitro)	Effective against dental pathogens	Mechanical strength; flexibility	Preclinical
3D-printed porous structures	35% higher BIC at 6 months (animal model)	Dependent on additional modifications	Patient-specific designs	Emerging clinical use
pH-responsive smart coatings	On-demand therapeutic release	99% antibacterial rate at pH 5.0 (in vitro)	Infection-triggered response	Laboratory stage
Antimicrobial peptides (GL13K)	Compatible with osseointegration	3-log reduction in biofilm (in vitro)	Low resistance potential	Preclinical
Bioactive glass (45S5)	BIC 57%–70% in animal models	Antibacterial via ion release	Bone bonding; ion exchange	Limited clinical use
Atomic layer deposition	Enhanced BIC with ZrO_2_ (animal model)	Inhibits MRSA without antibiotics	Conformal coating; atomic control	Preclinical
MOF-based platforms (ZIF-8)	Enhanced osteogenic gene expression	Synergistic Zn^2+^ + ROS generation	High porosity; tunable release	Laboratory stage

Technologies are grouped by mechanistic class. For each, the table summarizes the reported osseointegration enhancement, with the level of evidence (in vitro, animal model, or meta-analysis of animal data) indicated in parentheses, the antimicrobial effect, the key advantages, and the current stage of clinical translation. Quantitative values derived from animal histomorphometry are identified as such and are not interchangeable with clinical endpoints. All content is compiled by the authors from the cited primary literature; no previously published table is reproduced and no permissions are required.

SLA: sandblasted and acid-etched; TiO_2_: titanium dioxide; 3D: three-dimensional; ZrO_2_: zirconia; MOF: metal–organic framework; ZIF-8: zeolitic imidazolate framework-8; ROS: reactive oxygen species; MD: mean difference; BIC: bone-to-implant contact; OCN: osteocalcin; MRSA: methicillin-resistant *Staphylococcus aureus*.

### Limitations

This is a narrative review and carries the limitations inherent to that design. The literature search, although systematic in its construction and reported in full in the Scope, aim, and search strategy section, was not registered, and study selection was not governed by a prespecified protocol; selection bias in favor of positive and mechanistically interesting findings cannot be excluded. No formal risk-of-bias assessment was applied to individual studies, and no quantitative pooling was performed, so the quantitative values reported throughout are presented as they appear in the source publications and are not adjusted for study quality or heterogeneity. Restriction to English-language, full-text, peer-reviewed sources introduces language and publication bias, and negative or null results in surface engineering are systematically underreported. Much of the preclinical evidence derives from heterogeneous in vitro models and animal models whose bone metabolism, loading conditions, oral microbiota, and healing kinetics differ from those of humans, and effect sizes from animal histomorphometry, including the bone-to-implant contact differences cited in Table 1, should not be extrapolated to clinical endpoints. Surface characterization is reported inconsistently across the primary literature, with roughness frequently described by a single parameter and wettability reported without the storage interval, which limits comparability between studies and, for contact angle, can be positively misleading. Finally, for most of the emerging technologies reviewed here, including stimuli-responsive coatings, 4D-printed constructs, graphene derivatives, and theranostic surfaces, no long-term clinical data exist, and the conclusions drawn about them are necessarily provisional.

## Conclusions

The field of implant surface modification has evolved dramatically from Brånemark's original machined titanium implants to today's sophisticated multifunctional architectures. Contemporary nanoengineered, bioactive, antimicrobial, and stimuli-responsive surfaces address fundamental limitations through precisely engineered interactions with cells, tissues, and microorganisms (Table 1). Preclinical evidence consistently indicates that next-generation surfaces enhance osseointegration and reduce microbial colonization, and early clinical data are encouraging, but comparative clinical evidence for improved outcomes in patients with compromised conditions remains limited. However, substantial translational gaps persist between laboratory innovation and routine clinical adoption. Bridging these gaps requires sustained collaboration among materials scientists, biologists, clinicians, and regulatory authorities, supported by long-term comparative studies, harmonized standards for advanced multifunctional surfaces, and economic models that capture full life-cycle value. Whether these surfaces ultimately deliver longer-lasting implants and more individualized care will be determined by long-term clinical evidence that is not yet available.

## Data Availability

Data sharing is not applicable to this article, as no new data were created or analyzed in this study.
